# Synthesis and Cytotoxicity Studies of Titanocene C Analogues

**DOI:** 10.1155/2008/754358

**Published:** 2007-10-01

**Authors:** Megan Hogan, James Claffey, Eoin Fitzpatrick, Thomas Hickey, Clara Pampillón, Matthias Tacke

**Affiliations:** Conway Institute of Biomolecular and Biomedical Research, The UCD School of Chemistry andChemical Biology, Centre for Synthesis and Chemical Biology (CSCB), University College Dublin, Belfield, Dublin 4, Ireland

## Abstract

From the carbolithiation of 6-N,N-dimethylamino fulvene **(3)** and 2,4[bis(N,N-dimethylamino)methyl]-N-methylpyrrolyl lithium **(2a)**, N-(N′,N′-dimethylaminomethyl)benzimidazolyl lithium **(2b)**, or p-(N,N-dimethylamino)methylphenyl lithium **(2c)**, the corresponding lithium cyclopentadienide intermediate **(4a–c)** was formed. These three lithiated intermediates underwent a transmetallation reaction with TiCl_4'_ resulting in N,N-dimethylamino-functionalised titanocenes **5a–c**. When these titanocenes were tested against a pig kidney epithelial cell line (LLC-PK), the ${\text{IC}}_{50}$ values obtained were of 23, and 52 μM for titanocenes **5a** and **5b**, respectively. The most cytotoxic titanocene in this paper, **5c** with an ${\text{IC}}_{50}$ value of 13 μM, was found to be approximately two times less cytotoxic than its analogue Titanocene **C** (${\text{IC}}_{50}=5.5$ μM) and almost four times less cytotoxic than cisplatin, which showed an ${\text{IC}}_{50}$ value of 3.3 μM when tested on the LLC-PK cell line.

## 1. INTRODUCTION

Titanium-based reagents have significant potential against solid tumors. Budotitane ([cis-diethoxybis(1-phenylbutane-1,3-dionato)titanium(IV)]) looked very promising during its preclinical evaluation, but did not go beyond Phase I clinical trials, although a Cremophor EL-based formulation was found for this rapidly hydrolysing molecule [1]. Much more robust in this aspect of hydrolysis is titanocene dichloride (Cp_2_TiCl_2_), which shows medium antiproliferative activity in vitro but promising results in vivo [2, 3]. Titanocene dichloride reached clinical trials, but the efficacy of Cp_2_TiCl_2_ in Phase II clinical trials in patients with metastatic renal cell carcinoma [4] or metastatic breast cancer [5] was too low to be pursued.

More recently, novel methods starting from fulvenes [6–17] and other precursors [18–20] allow direct access to highly substituted titanocenes via reductive dimerisation, carbolithiation, or hydridolithiation of the fulvene followed by transmetallation in the last two cases.

**Figure 1:**
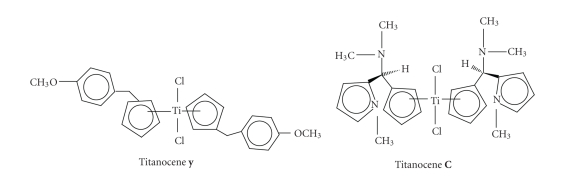
Structure of Titanocenes **Y** and **C**.

Titanocene **Y** was obtained using hydridolithiation of fulvene, and it showed an IC_50_ value of 21 *μ*M [12]. The antiproliferative activity of Titanocene **Y** has been studied in 36 human tumor cell lines [21] and in explanted human tumors [22]. These in vitro and ex vivo experiments showed that prostate, cervix, and renal cell cancers are prime targets for these novel classes of titanocenes, whereas the IC_50_ values for the breast cancer cell lines were very promising as well. These results were underlined by first mechanistic studies concerning the effect of these titanocenes on apoptosis and the apoptotic pathway in prostate cancer cells [23]. Furthermore, first animal studies have been published recently, reporting the successful treatment of xenografted Ehrlich's ascites tumor in mice with an ansa-titanocene [24] and xenografted Caki-1 tumors with Titanocene **Y** [25], showing that the effect of Titanocene **Y** against xenografted Caki-1 tumors in mice was superior to cisplatin. The structure of Titanocene **Y** is shown in Figure 1.

So far, our most cytotoxic titanocene, Titanocene **C** (bis-(N,N-dimethylamino-2(N-methylpyrrolyl)methylcyclo pentadienyl) titanium (IV) dichloride, was obtained through carbolithiation of fulvenes, which has been published recently [26]. It has an IC_50_ value of 5.5 *μ*M when tested on the LLC-PK cell line. Its structure is shown in Figure 1. This meant significant progress, since Cp_2_TiCl_2_ exhibits an IC_50_ value of only 2000 *μ*M against LLC-PK, which explains partly the failed Phase II clinical trials against renal cell carcinoma. The main idea behind the research presented in this paper was to improve the cytotoxicity of Titanocene **C** by adding extra dimethylamino groups using the well-established Mannich reaction. Within this paper, we present a new series of chiral titanocenes, their synthesis, and preliminary cytotoxicity studies.

## 2. EXPERIMENTAL

### 2.1. General conditions

Titanium tetrachloride (1.0 M solution in toluene) and butyl lithium (2.0 M solution in pentane) were obtained commercially from Aldrich Chemical Co. (Wis, USA). THF was dried over Na and benzophenone, and it was freshly distilled and collected under an atmosphere of argon prior to use. Manipulations of air and moisture sensitive compounds were done using standard Schlenk techniques under an argon atmosphere. NMR spectra were measured on either a Varian 300 or a 400 MHz spectrometer. Chemical shifts are reported in ppm and are referenced to TMS. IR spectra were recorded on a Perkin Elmer Paragon 1000 FT-IR Spectrometer employing a KBr disk. UV/Vis spectra were recorded on a Unicam UV4 Spectrometer, while CHN analysis was done with an Exeter Analytical CE-440 Elemental Analyser.

### 2.2. Synthesis

6-(N,N-dimethylamino) fulvene **(3)** was synthesised according to the already published procedure [27].

**Synthesis of bis-(3-[2,4-di(N,N-dimethylamino)methyl]- N-methylpyrrolyl-(N,N-dimethylamino)-methyl-cyclopentadienyl) titanium (IV) dichloride, {*η* ^5^–C_5_H_4_–CH[N(CH_3_)_2_][C_4_H_2_(CH_2_–N(CH_3_)_2_)_2_N(CH_3_)]}_2_TiCl_2_ (5a)
**

To a Schlenk flask with 2,4[bis(N,N-dimethylamino) methyl]-N-methyl pyrrole (1.61 g, 8.25 mmol), 20 ml of THF were added until a transparent solution was formed, while stirring at room temperature. The solution was cooled down to −78°C for 15 minutes and 4.8 mL (8.25 mmol) of butyl lithium were added. The solution was allowed to warm up to 0°C for 20 minutes, resulting in the formation of the yellow lithium intermediate.

In a second Schlenk flask, 6-(N,
N-dimethylamino) fulvene (1.00 g, 8.25 mmol) was dissolved in THF, and the resultant orange solution was added via cannula at −78°C to the Schlenk flask containing the lithiated intermediate. The reaction mixture was then allowed to warm up to room temperature and left stirring for 40 minutes. Titanium tetrachloride (4.1 ml, 4.13 mmol) was added afterwards in situ at room temperature, and the mixture was refluxed for 20 hours. Subsequently, the solvent was removed under vacuum, resulting in the formation of a dark brown oil that was dissolved in dichloromethane and filtered through Celite to remove the LiCl. The black filtrate was filtered additionally twice by gravity filtration. The solvent was removed under reduced pressure forming a shiny dark red solid, which was washed with 20 ml of pentane and then dried in vacuo (1.44 g, 1.93 mmol, 46.8% yield).

^1^H NMR (*δ* ppm CDCl_3_, 400 MHz): 6.36 (m, 8 H, C_5_ H_4_); 6.05 (s, 2 H, [C_4_ H(CH_2_–N(CH_3_)_2_)_2_N(CH_3_)]); 3.8–2.6 (m, 14 H, [C_4_H(CH_2_–N(CH_3_)_2_)_2_N(CH_3_)], [C_4_H(CH_2_–N(CH_3_)_2_)_2_N(CH_3_)], [C_4_H(CH_2_–N(CH_3_)_2_)_2_N(CH_3_)]); 2.36 (s, 26 H, C_4_ H(CH_2_–N(CH_3_)_2_)_2_N(CH_3_)).

^13^C NMR (*δ* ppm CDCl_3_, 125 MHz, proton decoupled): 138, 135, 132, 126, 121, 119, 108 [C_5_H_4_ and C_4_H(CH_2_–N(CH_3_)_2_)_2_N(CH_3_)]; 52 [C_5_H_4_–CH–(N(CH_3_)_2_)(C_4_H(CH_2_–N(CH_3_)_2_)_2_N(CH_3_)]; 34, 32 [N(CH_3_)_2_ and C_4_H(CH_2_–N(CH_3_)_2_)_2_N(CH_3_)].

IR absorptions (cm^−1^ KBr): 3414, 2917, 2769, 1620, 1466, 1382, 1018.

Anal. Calc. for C_38_H_62_N_8_TiCl_2_: C, 60.87; H, 8.35; N, 14.95; Cl, 9.46; Found: C, 59.80; H, 8.29; N, 14.18; Cl, 9.45.

UV-Vis (CH_2_Cl_2_): *λ* 244 nm (ɛ 10 833), *λ* 330 nm (ɛ 12 996), $\lambda_{\text{max}}$ 510 nm (weak).

**Synthesis of bis-[(N,N-dimethylaminomethyl- 2-benzimidazolyl)($N^{\prime },N^{\prime }$dimethylamino) methylcyclopentadienyl]titanium (IV) dichloride, {*η* ^5^–C_5_H_4_–CH[N(CH_3_)_2_][C_10_H_12_N_3_]}_2_TiCl_2_ (5b)
**

To a Schlenk flask with N-($N^{\prime },N^{\prime }$-dimethylaminomethyl) benzimidazol (1.45 g, 8.25 mmol), 20 ml of THF were added until a transparent solution was formed, while stirring at room temperature. The solution was cooled down to −78°C for 15 minutes and 14.0 ml (8.25 mmol, 1.7 M) of butyl lithium were added. The solution was allowed to warm up to 0°C for 20 minutes, resulting in the formation of the yellow lithium intermediate.

In a second Schlenk flask, 1.00 g (8.25 mmol) of 6-N,N-dimethylamino fulvene was dissolved in THF, and the resultant red solution was added via cannula at −78°C to the Schlenk flask containing the lithiated intermediate. The reaction mixture was then allowed to warm up to room temperature and left stirring for 40 minutes. Titanium tetrachloride (4.1 ml, 4.13 mmol) was added afterwards in situ at room temperature and the mixture was refluxed for 20 h. Subsequently, the solvent was removed under vacuum, resulting in the formation of a dark green oil that was dissolved in dichloromethane and filtered through Celite to remove the LiCl. The black filtrate was filtered additionally twice by gravity filtration. The solvent was removed under reduced pressure forming a shiny black solid, which was washed with 20 ml of pentane and then dried in vacuo (1.97 g, 2.77 mmol, 67.3% yield).

^1^H NMR (*δ* ppm CDCl_3_, 400 MHz): 7.80–7.82 [m, 4H, C_5_H_4_–CH[N(CH_3_)_2 _]–C–N–C–CH–CH–CH–CH–C–N (CH_2_N(CH_3_)_2_)]; 7.49–7.51 [m, 4 H, C_5_H_4 _–CH[N(CH_3_)_2_]–C–N–C–CH–CH–CH–CH–C–N(CH_2_N(CH_3_)_2_]; 6.42–6.71  [m, 8H, C_5_ H_4_–CH[N(CH_3_)_2_]–C_7_H_4_NN–CH_2-_N (CH_3_)_2_]; 4.85 [s, 4H, C_5_H_4_–CH[N(CH_3_)_2_]–C_7_H_4_NN–CH_2-_N (CH_3_)_2_]; 4.00 [m, 2H, C_5_H_4_–CH[N(CH_3_)_2_]–C_7_H_5_N_2_–CH_2-_N–(CH_3_)_2_]; 2.16, 2.23, 2.34 [s, 24 H, C_5_H_4_–CH  [N(CH_3_)_2_]–C_7_H_5_N_2_–CH_2-_N–(CH _3_)_2_].

^13^C NMR (*δ* ppm CDCl_3_, 125 MHz, proton decoupled): 144, 142, 136, 135, 132, 126, 123, 122, 121, 120, 119, 112[C_5_H_4_–CH[N(CH_3_)_2_]–C_7_H_5_N_2_–CH_2-_N–(CH_3_)_2_]; 69, 68,64,44,43,36,34,32,23,28,19,14,13[C_5_H_4_–CH[N(CH_3_)_2_]–C_7_H_5_N_2_–CH_2-_N–(CH_3_)_2_].

IR absorptions (cm^−1^ KBr): 3429, 2926, 1635, 1456, 1270, 1039, 861, 743.

Anal. Calc. for C_36_H_46_N_8_TiCl_2_: C, 60.93; H, 6.53; N, 15.79; Cl, 9.99 Found: C, 59.99; H, 6.52; N, 15.72; Cl, 9.99.

UV-Vis (CH_2_Cl_2_): *λ* 230 nm (ɛ 22 770), *λ* 402 nm (ɛ 2020), *λ* 499 nm (ɛ 210), $\lambda_{\text{max}}$ 523 nm (weak).

**Bis-(N,N-dimethylamino)-p-(N,N-dimethyl- amino)methylphenylcyclopentadienyl) titanium (IV) dichloride, {*η* ^5^-C_5_H_4_–CH[N(CH_3_)_2_][C_6_H_4_CH_2_N(CH_3_)_2_]}_2_TiCl_2_ (5c)
**

To a Schlenk flask with 0.37 g (1.73 mmol) p-(N,N-dimethylamino)methylphenylbromide, 14 ml of THF were added until a transparent solution was formed, while stirring at room temperature. The solution was cooled down to −78°C and 1.02 ml (1.73 mmol, 1.7 M) of t-butyl lithium were added. The solution was allowed to warm up to 0°C for 20 minutes, resulting in the formation of the yellow lithium intermediate.

In a second Schlenk flask, 0.21 g (1.73 mmol) of 6-(N,N-dimethylamino) fulvene was dissolved in THF, and the resultant red solution was added via cannula at −78°C to the Schlenk flask containing the lithiated intermediate. The reaction mixture was then allowed to warm up to room temperature and left stirring for 40 minutes. Titanium tetrachloride (0.86 ml, 0.86 mmol) was added afterwards in situ at room temperature and the mixture was refluxed for 20 h. Subsequently, the solvent was removed under vacuum, resulting in the formation of a dark brown oil that was dissolved in dichloromethane and filtered through Celite to remove the LiCl. The black filtrate was filtered additionally twice by gravity filtration. The solvent was removed under reduced pressure forming a shiny dark brown solid, which was washed with 150 ml of pentane and then dried in vacuo (0.45 g, 0.71 mmol, 82.7% yield).

^1^H NMR (*δ* ppm CDCl_3_, 300 MHz): 7.73–7.43 [m, 8H, C_6_ H_4_CH_2_N(CH_3_)_2_]; 6.70–6.40 [m, 8H, C_5_ H_4_]; 3.14 [s, 2H, C_5_H_4_–CH–(N(CH_3_)_2_(C_6_H_4_CH_2_N(CH_3_)_2_)]; 4.25–4.21 [s, 4H, C_5_H_4_–CH–(N(CH_3_)_2_(C_6_H_4_CH_2_N(CH_3_)_2_)]; 2.84–2.81 [s, 12H, C_5_H_4_–CH–(N(CH_3_)_2_(C_6_H_4_CH_2_N(CH_3_)_2_)]; 2.81–2.77 [s, 12H, C_5_H_4_–CH–(N(CH_3_)_2_(C_6_H_4_CH_2_N(CH_3_)_2_)].

^13^C NMR (*δ* ppm CDCl_3_, 125 MHz, proton decoupled): 146, 138, 136, 132, 131, 129, 127, 124, 120 [(C_6_H_4_ CH_2_N(CH_3_)_2_) and (C_5_H_4_)]; 61 [C_5_H_4_–CH–(N(CH_3_)_2_ (C_6_H_4_CH_2_N(CH_3_)_2_))]; 60 [(C_6_H_4_ CH_2_N(CH_3_)_2_)]; 42 [C_5_H_4_–CH–(N(CH_3_)_2_(C_6_H_4_CH_2_N(CH_3_)_2_))].

IR absorptions (cm^−1^ KBr): 3444, 3391, 2958, 2670, 2470, 1621, 1467, 1411, 1261, 1164, 1071, 1013, 943, 798.

Anal. Calc. for C_34_H_46_N_4_TiCl_2_: C, 64.90; H, 7.37; N, 8.91; Cl, 11.27 Found: C, 64.88; H, 7.36; N, 8.90; Cl, 11.27.

UV-Vis (CH_2_Cl_2_): *λ* 263 nm (*ɛ* 86 000), *λ* 275 nm (*ɛ* 94 000), *λ* 298 nm (*ɛ* 98 000), *λ* 320 nm (*ɛ* 108 000), *λ* 340 nm (*ɛ* 72 000), *λ* 390 nm (*ɛ* 35 000), $\lambda_{\text{max}}$ 455 nm (weak).

## 3. RESULTS AND DISCUSSION

### 3.1. Synthesis

**Scheme 1:**
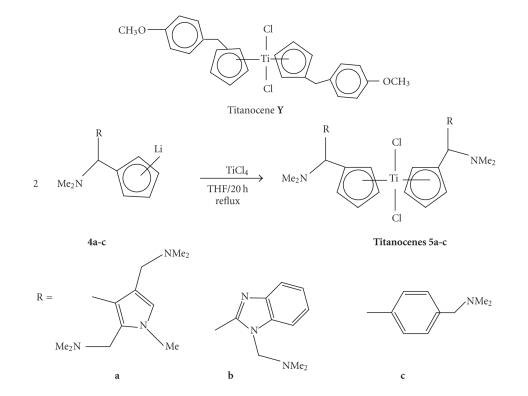
Synthesis of Titanocenes **5a–c**.

6-(N,N-dimethylamino) fulvene **(3)** was synthesised according to the already published procedure [27], and its structure is shown in Scheme 1.

The use of aryl lithium in the synthesis of other metallocenes is well known [28–30], and it has recently been used for the synthesis of chiral titanocene dichlorides [26].

This time, the carbolithiation method led to the synthesis of a new group of titanocenes that contain stereo centres **(5a–c)**.

The first step of the reaction consists of the formation of the functionalised lithium intermediates **(2a–c)** by reacting the corresponding heterocycles **(1a–c)** with *tert*-butyl lithium. Side reactions were avoided by cooling the reaction down to −78°C during the addition of *tert*-butyl lithium, and subsequent warming up to 0°C.

This step was followed by a nucleophilic addition of the lithiated intermediate to the double bond of 6-N,N-dimethylamino fulvene at −78°C. Then the reaction mixture was allowed to warm up to 0°C, resulting in the formation of the appropriately substituted lithium cyclopentadienyl intermediates **4a–c**. This reaction occurs with no stereo-selectivity, and the intermediates **4a–c** already contain a stereogenic carbon.

After stirring the reaction mixture for 40 minutes, two molar equivalents of **4a**, **4b** or **4c** underwent a transmetallation reaction when reacted with TiCl_4_ under reflux over 20 h in THF to give titanocenes **5a–c**.

The compounds obtained are shiny dark red solids. The synthesis of these compounds is shown in Scheme 1.

All three titanocenes shown in this paper have different isomers as seen in Figure 1. As a result of this, three different signals should be seen for every proton and carbon in the ^1^H and ^13^C NMR spectra. The R,R and S,S isomers are enantiomers and thus give identical NMR spectra, whereas for protons or carbons corresponding to the R,S (same as S,R) isomer, two signals can be observed as the environment of the two cyclopentadienyl rings is different. A relation of 2 : 1 : 1 for S,S and R,R, and the two signals for the S,R (or R,S) isomers can be observed in the integration pattern.

### 3.2. Cytotoxicity studies

**Figure 2:**
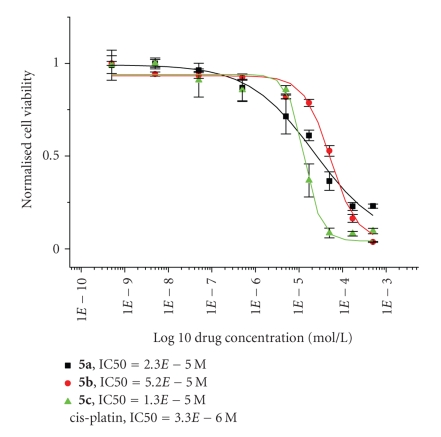
Cytotoxicity studies of Titanocenes **5a–c** against LLC-PK cells.

Preliminaryin vitro cell tests were performed on LLC-PK cells in order to compare the cytotoxicity of the compounds presented in this paper. This cell line was chosen based on their long-lasting growth behavior, similar to the one shown in carcinoma cells. It was obtained from the ATCC (american tissue cell culture collection) and maintained in Dulbecco's modified Eagle medium containing 10% (v/v) FCS (foetal calf serum), 1% (v/v) penicillin streptomycin, and 1% (v/v) L-glutamine. Cells were seeded in 96-well plates containing 200 *μ*l microtiter wells at a density of 5,000-cells/200 *μ*l of medium and were incubated at 37°C for 24 h to allow for exponential growth. Then the compounds used for the testing were dissolved in the minimal amount of DMSO (dimethylsulfoxide) possible and diluted with medium to obtain stock solutions of 5×10 ^−4^ M in concentration and less than 0.7% of DMSO. The cells were then treated with varying concentrations of the compounds and incubated for 48 hours at 37°C. Then the solutions were removed from the wells, the cells were washed with PBS (phosphate buffer solution), and fresh medium was added to the wells. Following a recovery period of 24 h incubation at 37°C, individual wells were treated with a 200 *μ*l of a solution of MTT (3-(4,5-dimethylthiazol-2-yl)-2,5-diphenyltetrazolium bromide) in medium. The solution consisted of 30 mg of MTT in 30 ml of medium. The cells were incubated for 3 hours at 37°C. The medium was then removed and the purple formazan crystals were dissolved in 200 *μ*L DMSO per well. Absorbance was then measured at 540 nm by a Wallac Victor (Multilabel HTS Counter) Plate Reader. Cell viability was expressed as a percentage of the absorbance recorded for control wells. The values used for the dose response curves of Figure 2 represent the values obtained from four consistent MTT-based assays for each compound tested.

As seen in Figure 2, Titanocenes **5a–c** showed an IC_50_ value of 23, 52, and 13 *μ*M, respectively.

When compared to unsubstituted titanocene dichloride (IC_50_ value = 2000 *μ*M), titanocene **5c** shows a major decrease in magnitude in terms of the IC_50_ value, and approximately a fourfold increase in magnitude with respect to cisplatin itself (IC_50_ value = 3.3 *μ*M). However, titanocene **5c** shows a decrease in cytotoxicity with respect to Titanocene **C**. The increased polarity of the new titanocenes together with an increase in size might be the cause of the decrease in cytotoxicity shown.

### 3.3. Structural DFT discussion

Despite our efforts to crystallise these three titanocenes, no crystal structures were obtained. This might be explained by the existence of different isomers in the racemic mixture. In order to overcome this problem, density functional theory (DFT) calculations were carried out for titanocene **5c** at the B3LYP level using the 6-31G** basis set [31], and compared with that of Titanocene **C** [26].

**Scheme 2:**
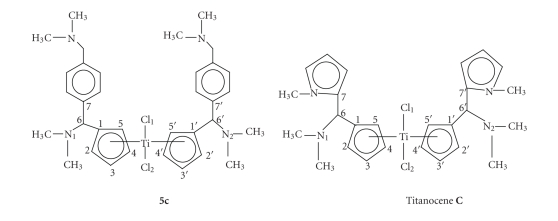
Numbering scheme of **5c** and Titanocene **C **for the structural DFT discussion of **5c**.

**Figure 3:**
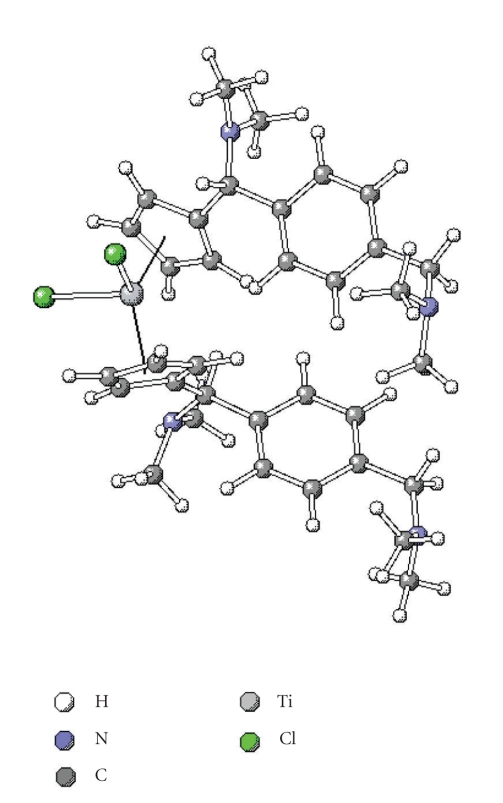
DFT calculated structure of **5c**.

Selected bond lengths of the optimised structure of titanocenes **5a** and **C** are listed in Table 1 (for atom numbering, see Scheme 2). The calculated structure of **5c** is presented in Figure 3.

The length of the bond between the metal centre and the cyclopentadienyl carbons is slightly different for the different Cp rings (251.3 and 248.5 pm, resp.). The same applies for the carbon-carbon bonds of the cyclopentadienyl rings with bond lengths between 140.2 and 143.4 pm.

The bond length between the methylic carbon centre and the carbon centre of the Cp group is of 152.2 and 152.0 pm, respectively. As well, the length of the bond between the methylic carbon and the nitrogen of the dimethylamino group is almost identical in all cases, and between 152.0 and 148.2 pm, respectively. The steric impediment of the dimethylamino groups attached to the methylic carbons causes a lengthening of the bond, in order to relieve the resultant steric strain.

The Cl–Ti–
Cl angle was calculated to be 95.1°. The angle formed between C_1_ and C_1′_, the respective methylic carbons (C_6_ or C_6*′*_), and C_7_ or C_7*′*,_ respectively, was of 11.42° in both cases, and almost identical to the one formed between each nitrogen of the dimethylamino group, C_6_ or C_6*′*_, and C_1_ and C_1*′*,_ respectively.

The DFT calculated structure of **5a** was then compared to the calculated structure of its mono-N-methylpyrrolyl-substituted analogue, Titanocene **C** [26]. In this complex, the length of the bond between the titanium centre and the two Cl atoms appeared to differ in only 1 pm approximately from the one found for **5c**, and of 234.9 and 236.1 pm, respectively. The same applies to the bond length between the N_1_ or N_2_ and C_6_ or C_6*′*,_ respectively, and to the length of the bond between the Cp carbon atoms and the titanium centre.

The Cl–Ti–
Cl angle in Titanocene **C** is very similar to the one calculated for **5c**, and of 94.9°, and so is the angle formed between the titanium centre and the centre of the Cp rings (with a difference of 0.3°).

Selected bond lengths from the calculated DFT structure of Titanocene **C** are listed in Table 1, while atom numbering can be seen in Scheme 2.

The cartesian coordinates for the DFT optimised structure of titanocene 5C can be observed in the supplementary material, where the energy of optimisation is also found.

## 4. CONCLUSIONS AND OUTLOOK

The carbolithiation of 6-(N,N-dimethylamino) fulvene with Mannich-functionalised lithiated species followed by transmetallation offers a general way into the synthesis of new chiral N,N-dimethylamino-functionalised metallocenes. Derivative **5c** exhibits an impressive cytotoxicity with an IC_50_ value of 13 *μ*M against LLC-PK cells, but is slightly less active with respect to Titanocene **C** that shows the highest cytotoxicity for a published titanocene so far.

## Figures and Tables

**Table 1:** Selected bond lengths from the DFT-calculated structure of **5c** and DFT-calculated structure of Titanocene **C**.

	DFT structure **(5c)**	DFT structure Titanocene **C**
	Bond length (pm)	Bond length (pm)
Ti−C_1_	251.3	250.4
Ti−C_2_	242.9	242.8
Ti−C_3_	244.2	240.0
Ti−C_4_	240.4	237.4
Ti−C_5_	243.2	242.9
Ti−C_1*′*_	248.5	247.8
Ti−C_2*′*_	239.1	239.0
Ti−C_3*′*_	234.2	233.1
Ti−C_4*′*_	245.6	243.7
Ti−C_5*′*_	250.2	249.3
C_1_−C_2_	143.4	143.2
C_2_−C_3_	141.6	141.5
C_3_−C_4_	141.2	141.3
C_4_−C_5_	142.3	142.3
C_5_−C_1_	141.4	141.4
C_1*′*_−C_2*′*_	141.4	141.4
C_2*′*_−C_3*′*_	142.2	142.4
C_3*′*_−C_4*′*_	142.3	142.2
C_4*′*_−C_5*′*_	140.2	140.2
C_5*′*_−C_1*′*_	143.0	143.0
C_1_−C_6_	152.2	152.2
C_1*′*_−C_6*′*_	152.2	152.0
C_6_−C_6*′*_	559.5	559.5
C_6_−C_7_	151.5	152.0
C_6*′*_−C_7*′*_	152.0	151.5
C_6_−N_1_	152.0	148.3
C_6*′*_−N_2_	149.7	148.4
Ti−Cl_1_	240.5	234.9
Ti−Cl_2_	234.1	236.1
